# Myosin VI facilitates connexin 43 gap junction accretion

**DOI:** 10.1242/jcs.199083

**Published:** 2017-03-01

**Authors:** Bennett J. Waxse, Prabuddha Sengupta, Geoffrey G. Hesketh, Jennifer Lippincott-Schwartz, Folma Buss

**Affiliations:** 1 Cell Biology and Metabolism Program, Eunice Kennedy Shriver National Institute of Child Health and Human Development, Bethesda, Maryland 20892, USA; 2 Cambridge Institute for Medical Research, University of Cambridge, Cambridge CB2 2XY, UK; 3 Mount Sinai Hospital, Lunenfeld-Tanenbaum Research Institute, Toronto, Ontario M5G 1X5, Canada

**Keywords:** Connexin, Endocytosis, Gap junction, Myosin

## Abstract

In this study, we demonstrate myosin VI enrichment at Cx43 (also known as GJA1)-containing gap junctions (GJs) in heart tissue, primary cardiomyocytes and cell culture models. In primary cardiac tissue and in fibroblasts from the myosin VI-null mouse as well as in tissue culture cells transfected with siRNA against myosin VI, we observe reduced GJ plaque size with a concomitant reduction in intercellular communication, as shown by fluorescence recovery after photobleaching (FRAP) and a new method of selective calcein administration. Analysis of the molecular role of myosin VI in Cx43 trafficking indicates that myosin VI is dispensable for the delivery of Cx43 to the cell surface and connexon movement in the plasma membrane. Furthermore, we cannot corroborate clathrin or Dab2 localization at gap junctions and we do not observe a function for the myosin-VI–Dab2 complex in clathrin-dependent endocytosis of annular gap junctions. Instead, we found that myosin VI was localized at the edge of Cx43 plaques by using total internal reflection fluorescence (TIRF) microscopy and use FRAP to identify a plaque accretion defect as the primary manifestation of myosin VI loss in Cx43 homeostasis. A fuller understanding of this derangement may explain the cardiomyopathy or gliosis associated with the loss of myosin VI.

## Abstract (precis)

**Highlighted Article:** Myosin VI localizes to gap junctions in a variety of cell types and tissues, where this motor regulates gap junction plaque size through interaction with cortical actin.

## INTRODUCTION

Cardiac function depends on the precise conduction of electrical activity throughout the myocardium, and disturbances to this highly coordinated process are the hallmark of acquired heart disease. Gap junctions (GJs) are semi-crystalline arrays of double-membrane-spanning intercellular channels that facilitate action potential propagation by electrochemically coupling neighboring cardiomyocytes. The basic subunit of the gap junction is the connexin, of which there are over 21 types expressed to variable degrees in nearly all human cell types (Dbouk et al., 2009; Söhl and Willecke, 2004). Of these, connexin 43 (Cx43; also known as GJA1) is the most widely expressed and it is the primary gap junction-forming protein in the ventricular myocardium (Laird, 2006; Severs et al., 2008). Indeed, derangement of the intercalated disc and Cx43 has been observed in pathological states, including idiopathic dilated cardiomyopathy, hypertrophy and acute cardiac ischemia (Kostin et al., 2003, 2004; Sepp et al., 1996; Smith et al., 1991). As such, Cx43 expression, localization and post-translational modifications are the subject of continued investigation.

To form a gap junction, connexin monomers are post-translationally inserted into the endoplasmic reticulum (ER) and oligomerized into hexameric transmembrane channels called connexons in the Golgi (Musil and Goodenough, 1993). By way of the secretory pathway, connexons are delivered to the plasma membrane, which they move within until they dock with connexons from an apposing cell (Lauf et al., 2002). Gap junctions have long been isolated as double membrane structures, precluding connexon separation and suggesting that removal of the intercellular junction requires endocytosis and exocytosis of both membranes in the form of an annular gap junction (AGJ) (Ghoshroy et al., 1995; Jordan et al., 2001). Ultimately, internalized AGJs are degraded by autophagy in a ubiquitin-dependent manner (Bejarano et al., 2012; Fong et al., 2012). Knowledge of this unidirectional pathway has long been accompanied by the fact that Cx43 exhibits a surprisingly short half-life for a junctional protein (ranging from 1.5–5 h), and while rapid turnover facilitates continuous remodeling, it also leaves connexins particularly susceptible to trafficking defects (Berthoud, 2004; Fallon and Goodenough, 1981; Laird et al., 1991). Ultimately, understanding of molecular mechanisms that govern the delicate balance of gap junction formation and internalization is paramount to the maintenance of intercellular communication.

Gap junction regulation is largely controlled by protein–protein interactions and post-translational modifications involving the Cx43 C-terminal tail. Plaque formation requires phosphorylation by casein kinase 1 and stabilization depends on the interaction of the Cx43 C-terminus with the actin-binding scaffolding protein ZO-1 (also known as TJP1) (thereby linking tight and GJs) (Cooper, 2002; Giepmans and Moolenaar, 1998). GJ plaque removal and decreased gap junction intercellular communication (GJIC) are associated with many processes, including phosphorylation by both Src and PKC, ubiquitylation by the protein ligase Nedd4, binding by the endocytic protein Eps15 and interaction with 14-3-3 proteins (Catarino et al., 2011; Girao et al., 2009; Smyth et al., 2014; Solan and Lampe, 2007; Toyofuku et al., 2001).

Historically, the clathrin machinery was also thought to be required for gap junction internalization. Clathrin and its alternative adaptor, Dab2, were detected on GFP-labeled Cx43 GJs by immunofluorescence [although the adaptor protein complex 2 (AP-2) was notably absent] and silencing of clathrin, AP-2, Dab2 or dynamin caused cells to harbor fewer AGJs (Gumpert et al., 2008; Piehl et al., 2007). Two tyrosine sorting signals and three phosphorylation sites within the Cx43 C-terminal tail were proposed to mediate this process (Fong et al., 2013, 2014). In addition, the Dab2-binding partner myosin VI was also found to localize to Cx43 GJs and it was suggested that myosin VI plays a role in the clathrin-mediated endocytosis of GJ plaques. However, these studies used the no insert (NI) isoform of myosin VI, which has no reported role in clathrin-mediated endocytosis. Furthermore, studies have questioned the role of clathrin in the internalization of double membrane GJs, suggesting that clathrin instead targets plasma membrane-localized connexons that have yet to form GJs (Johnson et al., 2013).

Myosin VI is the only unconventional myosin motor protein that moves toward the minus end of actin filaments, and it is expressed in four different splice variants in a tissue-specific manner. The large insert isoform is selectively expressed in polarized epithelial cells, where it is targeted to the apical domain to facilitate clathrin-mediated uptake of cell surface receptors (Ameen and Apodaca, 2007; Buss et al., 2001). In contrast, the no insert splice variant is widely expressed in most cell types and tissues, functioning in cargo sorting in the endocytic pathway, exocytosis, autophagy and the regulation of actin filament dynamics (Bond et al., 2011; Chibalina et al., 2007; Rogat and Miller, 2002; Tumbarello et al., 2012). At the plasma membrane, myosin VI functions at the interface between cell surface proteins and the cortical cytoskeleton, stabilizing cell–cell adhesion and anchoring apical hair cell membrane to the cortical actin meshwork (Geisbrecht and Montell, 2002; Heintzelman et al., 1994; Millo et al., 2004; Self et al., 1999). To mediate this variety of cellular functions, myosin VI interacts with a broad range of cargo adaptor proteins that bind to regions in the C-terminal cargo-binding tail with either an RRL sequence [binding partners GIPC1, TAX1BP1, NDP52 (also known as CALCOCO2) and optineurin] or with a WWY motif (binding partners TOM1, LMTK2 and Dab2) (Spudich et al., 2006; Tumbarello et al., 2013).

Interestingly, the loss of myosin VI in mice and humans manifests as two pathologies affiliated with connexin derangements: sensorineural deafness and hypertrophic cardiomyopathy (Avraham et al., 1995; Hegan et al., 2015; Mohiddin et al., 2004; Williams et al., 2013). In this study, we further investigate the association of myosin VI with Cx43 GJs in heart tissue and describe a new role for myosin VI in GJ homeostasis. Previous studies have separately reported myosin VI localization to GJ plaques in cell culture models and intercalated discs in heart sections (Karolczak et al., 2014; Piehl et al., 2007). We show that: (1) myosin VI recruitment to Cx43 GJs observed in native heart tissue extends to isolated cardiomyocytes; (2) myosin VI is not required for delivery of Cx43 to, or its movement within, the plasma membrane; and (3) loss of myosin VI instead impairs GJ plaque formation and downregulates intracellular communication.

## RESULTS

### Myosin VI localizes to GJs in heart tissue and primary cardiomyocytes

**Fig. 1.**
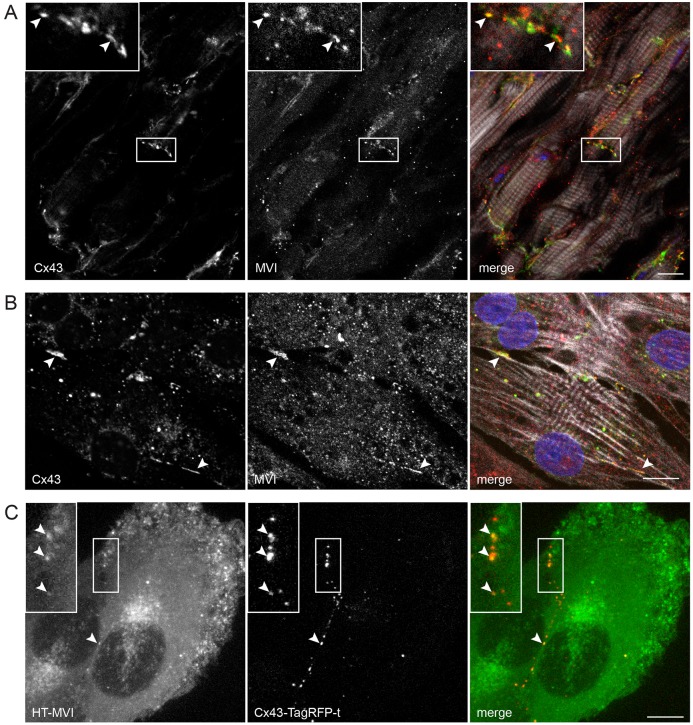
**Myosin VI localizes to Cx43 GJs.** (A) Heart sections and (B) isolated NRVMs were processed for confocal immunofluorescence microscopy to assess endogenous Cx43 (green) and myosin VI (MVI, red) colocalization. Nuclei were labeled with Hoechst 33342 (blue) and F-actin was labeled with Alexa-Fluor-647–phalloidin (white). (C) Live NRK cells stably expressing GFP–myosin-VI (green) and Cx43–TagRFP-t (red) were imaged on a Nikon spinning disk microscope. Arrowheads identify myosin VI localization to Cx43 plaques at intercellular junctions. Scale bars: 10 µm.

Given previous observations of myosin VI colocalization with its binding partner Dab2 on GJs in HeLa cells expressing GFP–Cx43 (Piehl et al., 2007), we first examined the subcellular localization of endogenous myosin VI and Cx43 in the adult mouse heart and neonatal rat ventricular myocytes (NRVMs). Whole frozen heart sections were fixed and analyzed by immunohistochemistry. We detected myosin VI throughout cardiac tissue, and it was enriched on Cx43 structures at the intercalated disc (Fig. 1A, arrowheads). We then isolated primary NRVMs and, by immunofluorescence, we confirmed that myosin VI strongly decorates Cx43 gap junction plaques (Fig. 1B, arrowheads). Unlike myosin VI, neither Dab2 nor clathrin localized to Cx43 GJs in our primary cardiomyocyte experiments using NRVMs (Fig. S1B,C). Western blotting confirmed that myosin VI is detected in whole-heart homogenates (Fig. S3A) and purified cardiomyocytes (Fig. S1A).

In addition to primary heart tissue, we also used normal rat kidney (NRK) cells, which endogenously express Cx43 and myosin VI, to confirm the colocalization of these proteins. Using retroviral transduction, we created cell lines stably expressing Cx43–TagRFP-t, in which we transiently expressed HaloTag (HT)-conjugated myosin VI. HT–myosin-VI strongly colocalized with Cx43–TagRFP-t-labeled GJs (Fig. 1C, arrowheads), thus supporting the localization of myosin VI at GJs observed in whole-heart sections and primary NRVMs.

### Myosin VI recruitment to Cx43 GJs requires the RRL binding motif

**Fig. 2.**
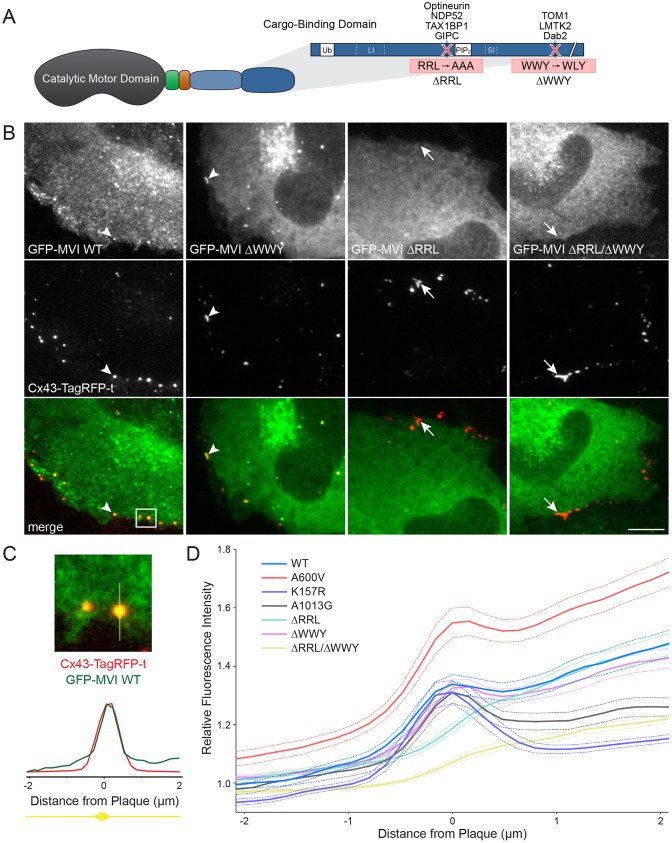
**Myosin VI targets to Cx43 GJs through the RRL motif in the myosin VI cargo-binding tail domain.** (A) Cartoon depicting the domain structure of myosin VI (MVI). Myosin VI contains a catalytic motor domain, a unique insert (green), an IQ calmodulin-binding motif (orange), a tail region containing a three-helix bundle and a single α-helix (SAH) domain (light blue), and a cargo-binding tail domain (dark blue). The cargo-binding tail contains protein interaction motifs (RRL and WWY) as well as binding regions for ubiquitin and phosphatidylinositol 4,5-bisphosphate. Alternative splicing can give rise to insertions of both a large and small insert (LI, SI). (B) NRK cells stably expressing Cx43–TagRFP-t (red) were transfected with full-length GFP–myosin-VI (green) constructs with point mutations, fixed and processed for confocal microscopy. Visually, colocalization was lost in the ΔRRL and ΔWWY/ΔRRL mutants (arrows) compared to the wild type (WT) and ΔWWY mutant (arrowheads). (C) 4-µm line plots for GFP and TagRFP-t fluorescence were then drawn across all Cx43 GJs (illustrated for the boxed region from B) toward the center of the GFP–myosin-VI -expressing cell. (D) Channel intensity for myosin VI (dashed lines indicate ±s.e.m.) was plotted for each GJ and averaged for at least 256 plaques in at least 30 cells for each mutant. Neither the ΔRRL nor the ΔRRL/ΔWWY myosin VI mutant showed a peak in intensity at Cx43 GJs. Scale bar: 10 µm.

Myosin VI is composed of a conserved catalytic domain and a C-terminal cargo-binding domain that contains ubiquitin- and lipid-binding domains and mediates binding to adaptor proteins via RRL and WWY motifs (Fig. 2A) (Chibalina et al., 2007; Morriswood et al., 2007; Sahlender et al., 2005; Tumbarello et al., 2012). To determine which binding domain and potential adaptor protein are required for myosin VI localization to Cx43 GJs, we transfected NRK cells stably expressing Cx43–TagRFP-t with GFP–myosin-VI full-length constructs (Fig. 2B) harboring amino acid substitutions known to disrupt these binding domains. Fixed cell fluorescence microscopy demonstrated that the ΔWWY mutant mimics wild type localization of myosin VI to Cx43 (arrowheads), while the loss of the RRL binding motif prevented myosin VI targeting to Cx43 (arrows).

To confirm this observation, line plots drawn through GJ plaques were quantified as depicted (Fig. 2C). Line plots were oriented with the GFP–myosin-VI-expressing cell to the right. While not included in Fig. 2B, motor domain (A600V and K157R) and ubiquitin-binding (A1013G) mutants were also tested for GJ recruitment. Myosin VI fluorescence intensity reached a local maximum at the Cx43 GJ plaque center (0 µm) in all plots except RRL mutants (Fig. 2D). These results clearly establish that myosin VI localization to Cx43 GJs does not depend on motor activity, the A1013 ubiquitin-binding site or the WWY-binding partners TOM1, LMTK2 or Dab2.

We then asked if the RRL-binding partners OPTN, TAX1BP1 or GIPC1 also localize to Cx43 GJs. NDP52 was omitted because rodents express a truncated isoform that lacks the myosin VI-binding zinc finger domain (Tumbarello et al., 2015). In fixed primary NRVMs, antibodies raised against OPTN, TAX1BP1 and GIPC1 failed to decorate Cx43 GJ plaques (Fig. S2A, arrowheads). We obtained the same result using NRK cells stably expressing Cx43–TagRFP-t and HT–myosin-VI transfected with GFP–OPTN, GFP–TAX1BP1 or GFP–GIPC1 (Fig. S2B, arrowheads). These results suggest that myosin VI targets to Cx43 either directly, via an unknown binding partner or ubiquitin binding through the MyUb domain – a recently described region that overlaps with the RRL binding region (He et al., 2016).

### Myosin VI depletion leads to a reduction in GJ plaque size

**Fig. 3.**
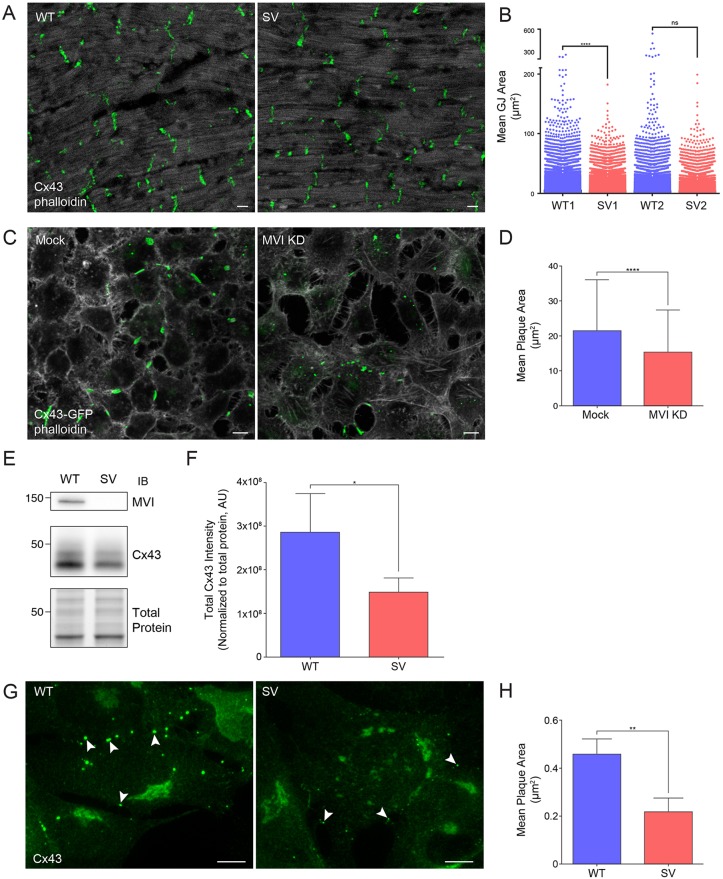
**The loss of myosin VI results in reduced GJ size.** (A) Whole-heart sections from wild-type (WT) and *sv* (SV) mice were processed for confocal immunofluorescence microscopy and immunostained for Cx43 (green) and stained for F-actin (white). (B) Cx43 GJ plaques were identified and quantified for two pairs of hearts (26,000–47,000 structures per heart). ****P<0.0001; ns, not significant. (C) Confluent monolayers of HeLa cells stably expressing Cx43–GFP (green) were transfected with non-targeting or myosin VI siRNA, stained for F-actin and imaged. (D) GJ plaque area was quantified using ImageJ (mean±s.d., *n*=3). ****P<0.0001. (E) Immunoblot for Cx43 and myosin VI in WT and *sv* MEFs. (F) Quantification of western blot Cx43 intensity normalized by Bio-Rad Stain-Free tryptophan labeling (total protein) (mean±s.d., *n*=5). *P<0.05. (G) WT and *sv* MEFs were processed for confocal immunofluorescence microscopy and immunostained for Cx43 (green). Arrowheads indicate GJ plaques in WT and SV cells. (H) Cx43 GJ plaque area was quantified using ImageJ. (mean±s.d., *n*=3). **P<0.01. Scale bars: 10 µm.

To investigate the ramifications of myosin VI loss on GJ maintenance and morphology, we isolated heart tissue from the *Snell's waltzer* (*sv*) myosin VI-null mouse. While blotting whole-heart homogenates showed only a subtle decrease in total Cx43 (Fig. S3A,B), a selective loss of Cx43 from the plasma membrane is observed by immunohistochemistry in fixed *sv* hearts. In the myosin VI-null heart, Cx43 still localized to the intercalated disc, but individual Cx43 structures were smaller (Fig. 3A). Quantification of all Cx43 structures in *sv* hearts (between 26,000 and 47,000 per heart) verified this reduction in average Cx43 area compared to wild-type hearts (Fig. 3B).

We also depleted myosin VI by using siRNA in RPE cells and HeLa cells stably expressing Cx43. As we observed for whole-heart homogenates, the knockdown of myosin VI in HeLa cells (Fig. S3C) did not lead to a marked change in total Cx43; however, fixed cell fluorescence showed a reduction in the Cx43–GFP-labeled GJ plaque size when myosin VI was silenced in HeLa stable cell lines (Fig. 3C). Quantification of all Cx43 GJ plaques from these images (Fig. 3D) confirmed that the average plaque size is reduced when myosin VI is silenced.

To further corroborate these observations, we isolated and immortalized mouse embryonic fibroblasts (MEFs) from wild-type and *sv* mice. Here, western blotting for Cx43 (Fig. 3E) showed a sizeable reduction in the amount of total Cx43 in *sv* MEFs (Fig. 3F). By immunofluorescence, we again observed a decrease in Cx43 GJ plaque size when myosin VI was lost (Fig. 3G). Quantification of these microscopy experiments verified this observation (Fig. 3H). These results indicate that the primary effect of myosin VI loss is a reduction in Cx43 GJ plaque size in heart tissue, HeLa cells and immortalized *sv* MEFs.

### Myosin VI depletion impairs GJ intercellular communication

**Fig. 4.**
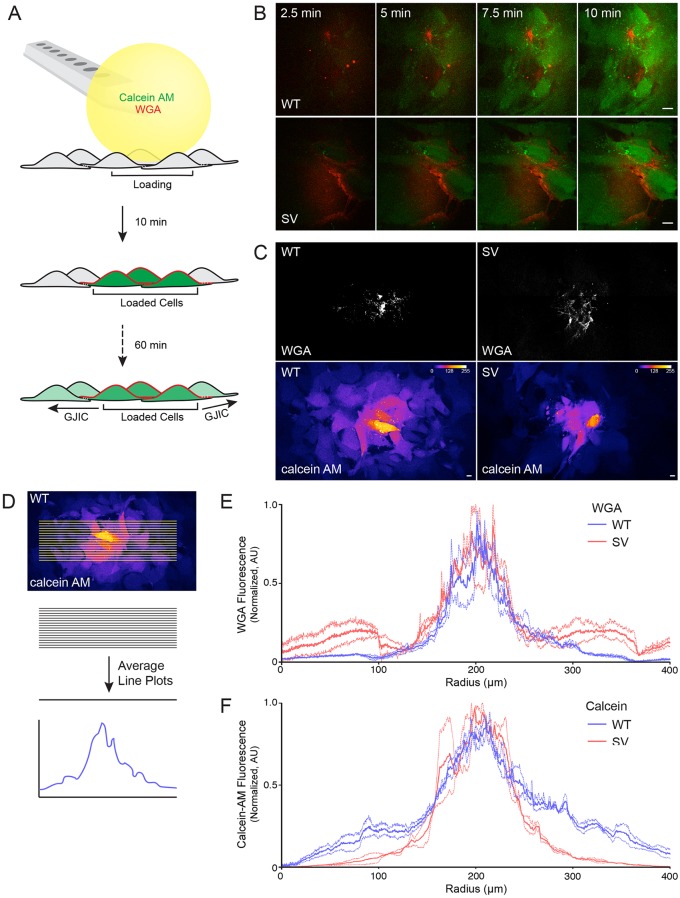
**Gap junction intercellular communication is reduced in myosin VI-null MEFs.** (A) Cartoon detailing an assay to measure GJIC. We used the BioPen microfluidic pipette to administer 20 µM calcein AM and 1 µg/ml WGA to monolayers of wild-type (WT) and *sv* (SV) MEFs for 10 min. The total spread of calcein AM through GJs was measured 60 min later. (B) Calcein AM (green) and WGA (red) loading was imaged using live-cell spinning disk microscopy over 10 min. (C) Tiled images surrounding the site of administration (WGA, grayscale) were obtained 60 min later to visualize the extent of transfer of calcein (Fire LUT). (D) Cartoon depicting the quantification method in which line scans were drawn across cell monolayers. A rotated image from C is re-shown for demonstrative purposes. Signal intensities across each sample were averaged and plotted for WGA–647 (E) and Calcein AM (F) (dashed lines indicate ±s.e.m., *n*=3). Scale bars: 10 µm.

We then asked whether the reduction in GJ plaque size is associated with a concomitant reduction in GJIC in myosin VI-null cells. For these experiments, we used the BioPen microfluidic pipette (Ainla et al., 2010) to generate a hydrodynamically confined fluid sphere to selectively load multiple cells with calcein AM (Fig. 4A), which converts into a GJ-permeable dye within cells. To demarcate the zone of calcein AM administration, 1 µg/ml wheat germ agglutinin conjugated to Alexa Fluor 647 (WGA–647) was added to a solution of 20 µM calcein AM and cells were loaded for 10 min (Fig. 4B). After 60 min, a tiled image of the administration site was obtained, showing that *sv* MEFs transfer substantially less calcein AM across a monolayer compared to wild-type cells (Fig. 4C). To quantify this difference, line plots were drawn across the calcein administration zone and the intensities were averaged for each channel (Fig. 4D). Line plots from multiple experiments were normalized, averaged and plotted for WGA–647 (Fig. 4E) to confirm equivalent administration of calcein AM. In contrast, calcein AM spread (Fig. 4F) was much lower in *sv* MEFs compared to wild-type cells.

**Fig. 5.**
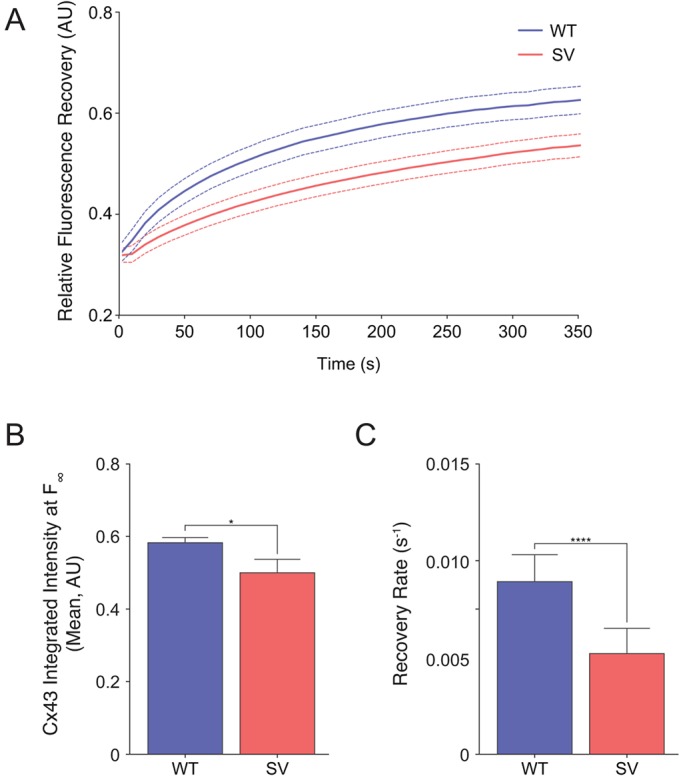
**Loss of myosin VI compromises GJ intercellular transport.** (A) Wild-type (WT) and *sv* (SV) MEFs were loaded with calcein AM and cells were photobleached for FRAP analysis. Fluorescence recovery as a function of time was plotted (mean, ±s.e.m. is indicated by the region between the dashed lines, *n*=3). (B) Quantification of fluorescence intensity at *t*, *F*_∞_ as calculated by linear regression analysis (mean±s.d., *n*=3). *P<0.05. (C) Quantification of recovery rate. (mean±s.d., *n*=3). ****P<0.0001.

To confirm these findings and to verify this new method, we also conducted fluorescence recovery after photobleaching (FRAP) experiments (Abbaci et al., 2007; Bastide et al., 1995) with wild-type and *sv* MEFs. In accordance with our micropipette loading assay, calcein AM fluorescence recovery as calculated by linear regression analysis (Santiquet et al., 2012) was reduced when myosin VI was lost (Fig. 5A). In this analysis, total fluorescence recovery (Fig. 5B) was decreased in the absence of myosin VI, but the more striking effect was a decrease in the rate of fluorescence recovery (Fig. 5C). These results indicate that the reduction in GJ size due to myosin VI loss corresponds with defective GJIC.

### Plaques accumulate more slowly without myosin VI, but not due to defective Cx43 forward trafficking

**Fig. 6.**
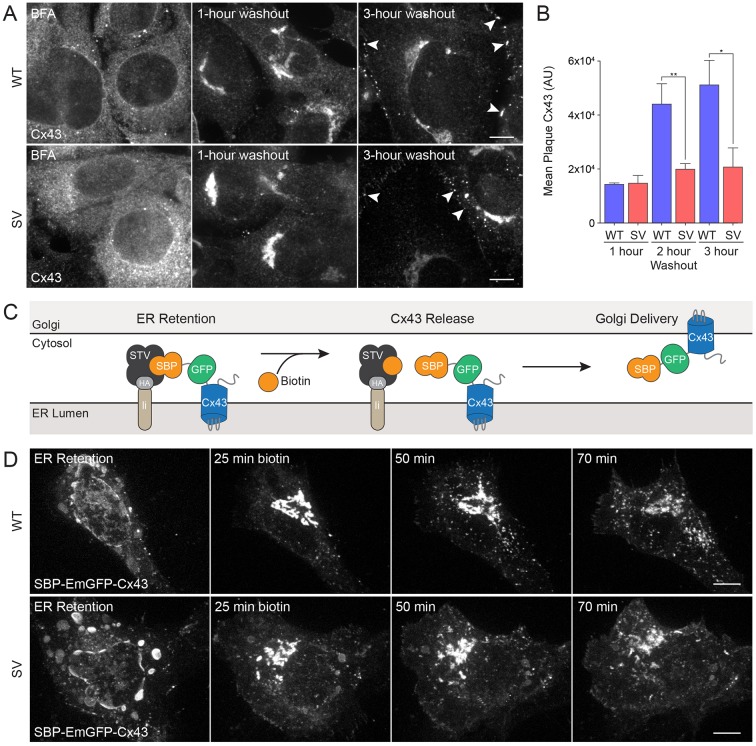
**Cx43 GJ plaque formation is reduced in myosin VI-null MEFs, but Golgi trafficking remains intact.** (A) Wild-type (WT) and *sv* (SV) MEFs were treated for 6.5 h with 5 µg/ml Brefeldin A (BFA). Cells were fixed after washout periods of 0, 1, 2 or 3 h, processed for confocal immunofluorescence microscopy and immunostained for Cx43 (grayscale) to identify GJ plaques (arrowheads). (B) GJ plaque area was quantified using ImageJ (mean±s.d., *n*=3). *P<0.05, **P<0.01. (C) Cartoon depicting the RUSH trafficking assay. Upon the addition of biotin to the medium, GFP–Cx43 fused to streptavidin-binding peptide (SBP–GFP–Cx43) is displaced from the ER retention hook (core streptavidin anchored to an invariant chain of the major histocompatibility complex, Ii–STV). SBP–GFP–Cx43 then freely traffics from the ER, through the Golgi, to the plasma membrane. (D) Wild-type and *Snell's waltzer* MEFs were transiently transfected with SBP–GFP–Cx43 – a bicistronic construct that also contains the ER hook. Biotin was added to the medium and cells were imaged on a Nikon spinning disk microscope to follow SBP–GFP–Cx43 trafficking. Scale bars: 10 µm.

Considering known roles for myosin VI in protein secretion, we next investigated whether the forward trafficking of Cx43 from the ER to the plasma membrane was delayed in myosin VI-depleted cells (Bond et al., 2011; Chibalina et al., 2010). We first analyzed the formation of endogenous GJ plaques in *sv* MEFs using a brefeldin A (BFA) forward trafficking assay. Treatment with BFA induces a reversible inhibition of COPI-mediated transport from the ER to the Golgi. Wild-type and *sv* MEFs were treated with 5 µg/ml BFA for 6.5 h, resulting in accumulation of Cx43 in the ER. BFA washout restored transport and cells were fixed 1, 2 or 3 h later. One hour after washout, the vast majority of Cx43 had left the ER and localized to the Golgi. Three hours after washout, Cx43 had trafficked to the cell surface and GJ plaques accumulated between cells (Fig. 6A). Interestingly, our results demonstrate that after restoring the secretory pathway with BFA washout, the intracellular GJ plaques grow at a slower rate and remain smaller in *sv* MEFs compared to wild-type MEFs (Fig. 6A,B).

To assess whether loss of myosin VI causes any delays in the trafficking of Cx43–GFP through the secretory pathway, we also used the retention using selective hooks (RUSH) system (Boncompain et al., 2012) to control and synchronize Cx43–GFP release from the ER, since the folding and maturation time inherent to GFP makes it difficult to observe the early secretory itinerary of Cx43–GFP. We created a Cx43 fusion protein tagged with an N-terminal GFP and streptavidin-binding protein (SBP–GFP–Cx43) that was expressed under the same promoter as core streptavidin anchored to the ER using the major histocompatibility complex (MHC) invariant chain (Ii). In this system, SBP–GFP–Cx43 is retained in the ER, but upon biotin addition, SBP–GFP–Cx43 freely traffics through the secretory pathway (Fig. 6C).

We first co-expressed HT–myosin-VI and SBP–GFP–Cx43 (Fig. S4A) in NRK cells to determine whether these proteins colocalized throughout SBP–GFP–Cx43 trafficking. Before the addition of biotin, SBP–GFP–Cx43 was confined to intracellular puncta that lacked myosin VI labeling. At 25 min after biotin addition, most SBP–GFP–Cx43 was confined to the Golgi, where no myosin VI was present. At 50 min minutes after biotin addition, SBP–GFP–Cx43-containing trafficking intermediates en route to the plasma membrane were observed, and these were also devoid of myosin VI (arrowheads, Fig. S4A).

These results suggest that myosin VI does not play a role in SBP–GFP–Cx43 forward trafficking, but to validate this observation, we conducted similar experiments in wild-type and *sv* MEFs (Fig. 6D). Upon biotin addition, ER-localized SBP–GFP–Cx43 trafficked to the Golgi, accumulating there within 25 min in both cell types (Fig. 6D). During this period, there appeared to be no qualitative difference in the trafficking of SBP–GFP–Cx43, suggesting myosin VI is not required for movement of Cx43 through the secretory pathway.

### Myosin VI contributes to Cx43 accretion at GJ plaques

**Fig. 7.**
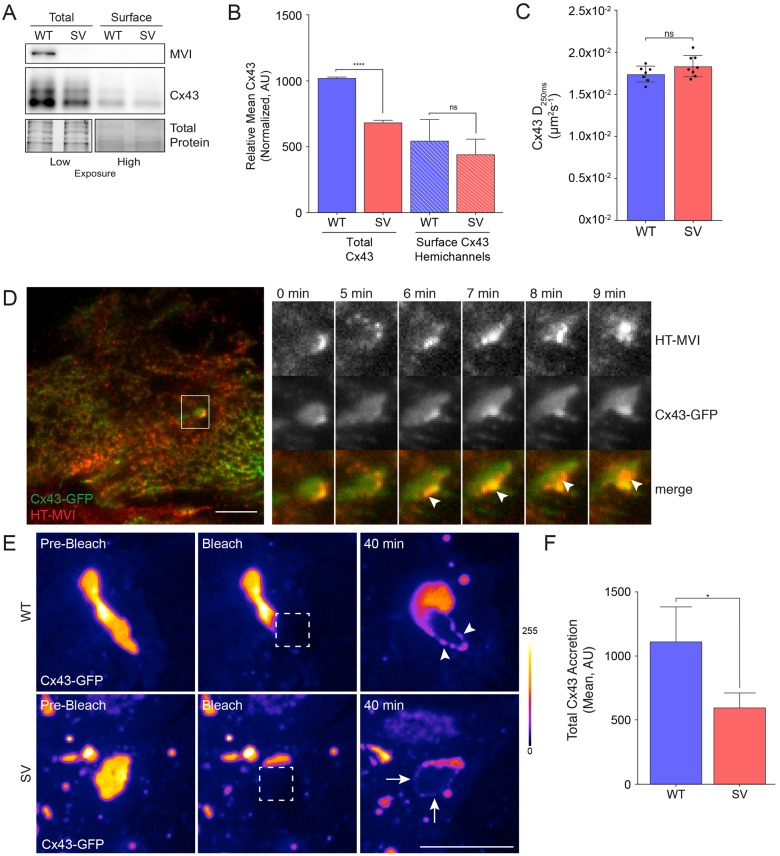
**Plasma membrane-localized Cx43 exhibits an accretion defect in myosin VI-null MEFs.** (A) The surface of wt and *sv* (SV) MEFs was biotinylated at 4°C and biotinylated proteins were isolated using NeurtrAvidin beads. Whole-cell lysates and biotinylation fractions were immunoblotted with an antibody against Cx43. MVI, myosin VI. (B) Quantification of western blot Cx43 intensity normalized by Bio-Rad Stain-Free tryptophan labeling (total protein) (mean±s.d., *n*=3). ****P<0.0001; ns, not significant. (C) Wild-type and *sv* MEFs were transfected with mEOS3–Cx43 and imaged on a Nikon TIRF microscope for single-particle-tracking photoactivation localization microscopy analysis. The diffusion coefficient of mobile mEOS3–Cx43 (Cx43 D_250ms_; mean±s.d.) was quantified for at least seven cells for each phenotype. (D) NRK cells stably expressing Cx43–GFP (green) and HT–myosin-VI were incubated with the JF_646_-HaloTag ligand (red) before imaging using TIRF microscopy to identify peripheral recruitment of myosin VI to Cx43 GJ plaques (arrowheads). (E) WT and *sv* MEFs were transfected with Cx43–GFP (Fire LUT) and imaged using a 3i Spinning Disk microscope for FRAP experiments. Square regions of Cx43 plaques were photobleached (dotted box) and imaged over 40 min to measure Cx43 accretion. WT MEF accretion (arrowhead) was greater than that observed in *sv* MEFs (arrows). (F) Cx43 accretion was quantified using ImageJ (mean±s.d., *n*=4). *P<0.05. Scale bars: 10 µm.

To further assess whether loss of myosin VI causes a defect in Cx43 trafficking to the cell surface, we quantified the plasma membrane-localized population of Cx43 connexons using a surface biotinylation assay in wild-type and *sv* MEFs. While total Cx43 was markedly reduced in *sv* MEFs, the amount of Cx43 connexons in the plasma membrane was not significantly different compared to wild-type MEFs (Fig. 7A,B). Since less Cx43 is incorporated into GJ plaques in *sv* MEFs compared to wild-type cells (see Fig. 3G,H), it follows that these cells contain a larger fraction of free Cx43 in the plasma membrane as compared to wild-type MEFs.

We next performed single-particle-tracking photoactivation localization microscopy (sptPALM) in wild-type and *sv* MEFs expressing a monomeric (m)EOS3–Cx43 fusion protein to test whether there were differences in the mobility of the free molecules in the plasma membrane of these cells. As shown in Fig. 7C, no difference in mEOS3–Cx43 mobility outside of plaques was seen in the two cell types, indicating myosin VI depletion does not affect free Cx43 movement in the plasma membrane (Fig. 7C).

We then addressed whether myosin VI serves a role in GJ plaque stability or biogenesis, since our localization studies show a strong enrichment of myosin VI on Cx43 GJs. We noticed that on rare occasions, Cx43 GJ plaques extend close to the coverslip. This allowed us to take advantage of the reductions in the out-of-focus light that total internal reflection fluorescence (TIRF) microscopy affords. Using this technique, we imaged NRK cells transfected with Cx43–GFP and HT–myosin-VI, observing that HT–myosin-VI localizes to the edge of GJ plaques (arrowhead, Fig. 7D; Movie 1). To investigate Cx43 GJ accretion (i.e. assembly into a GJ plaque), we used a photobleaching assay in wild-type or *sv* MEFs transfected with Cx43–GFP. A small portion of a large Cx43–GFP plaque was photobleached as indicated (Fig. 7E, dotted white box) and imaged 40 min later to test for recovery of Cx43–GFP fluorescence in this area. In wild-type MEFs, partial recovery at the plaque edge was observed (see arrowheads in WT panel), suggesting that free Cx43–GFP molecules were undergoing accretion at the plague edge over time. In *sv* cells, by contrast, much less Cx43–GFP was seen accumulating at plaque edges (Fig. 7E,F), suggesting a defect in the ability of Cx43 to be recruited into the plaque.

## DISCUSSION

**Fig. 8.**
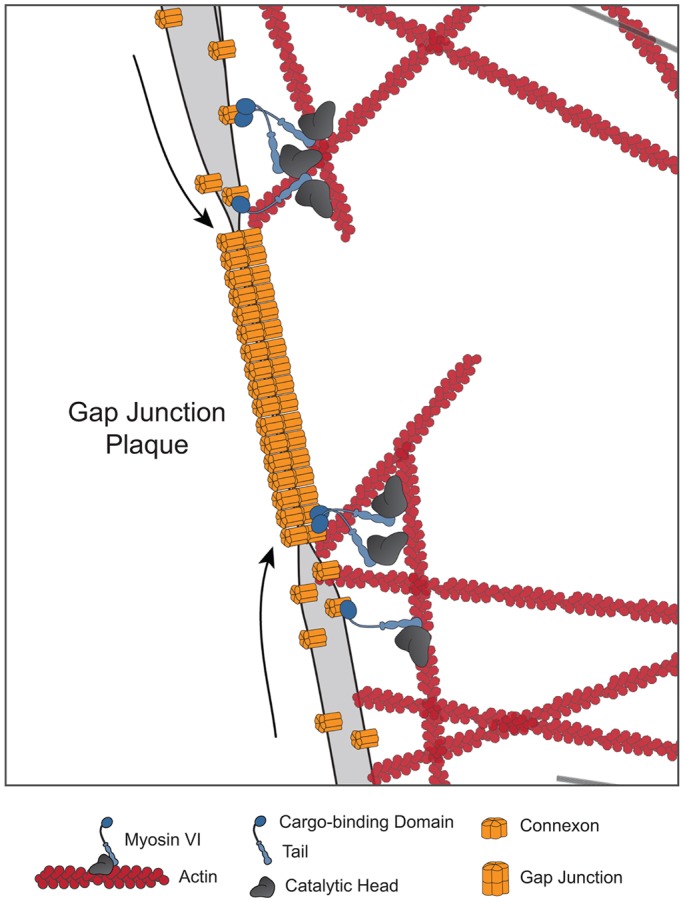
**Myosin VI facilitates Cx43 GJ accretion.** Our experiments showed that myosin VI did not localize together with Cx43 along the biosynthetic pathway, and that loss of myosin VI did not affect Cx43 delivery to or trafficking within the plasma membrane. We also did not observe clathrin and Dab2 localization colocalization with Cx43 at the plasma membrane. Instead, by using TIRF microscopy, we visualized myosin VI at the periphery of Cx43 GJ plaques, and by using FRAP, we observed an Cx43 accretion defect when myosin VI was lost. We hypothesize that it is at the GJ plaque edge that myosin VI aids in actin-mediated Cx43 accretion.

In this study, we used new and traditional assays of GJ trafficking and function to redefine numerous aspects of the role of myosin VI in Cx43 GJ homeostasis. First, we confirmed a previous report that endogenous myosin VI localizes to GJs in heart tissue (Karolczak et al., 2014) and we extended this finding to primary mouse cardiomyocytes and identified the RRL or MyUB binding motif as the basis of this localization. Second, we showed that the loss of myosin VI leads to a reduction in GJ plaque size not only in myosin VI siRNA HeLa knockdown cells but also in cardiac tissue and fibroblasts from the *Snell's waltzer* mouse. Third, we observed a concomitant reduction in GJ intercellular communication using a new method of selective calcein administration. Finally, we found that the observed reduction in GJ size was not due to the defective delivery of Cx43 to the cell surface via the biosynthetic pathway but instead was caused by impaired plaque accretion (Fig. 8).

Prior work has suggested that localization of myosin VI at GJ plaques is linked to its role in GJ internalization by a clathrin- and Dab2-mediated process (Piehl et al., 2007). While our work confirms myosin VI localization at GJs in primary cardiac tissues and isolated cardiomyocytes, it also suggests that this localization is not involved in GJ internalization. None of our cells showed clathrin or Dab2 localized at GJ plaques. Moreover, we found that WWY motif of myosin VI (which interacts with Dab2) is dispensable to GJ localization of myosin VI. Therefore, myosin VI function at GJs is unlikely to be related to clathrin-mediated endocytosis.

This conclusion is consistent with the fact that the cells used in the Piehl et al. study did not express the LI isoform of myosin VI that is involved in clathrin-mediated endocytosis (Aschenbrenner et al., 2003; Buss et al., 2001). The putative Dab2-binding motif (xPxY) that exists in the Cx43 tail, therefore, may be more important for Cx43 hemichannel rather than plaque endocytosis.

In our studies examining myosin VI recruitment to GJs, we identified a requirement of the RRL binding motif on myosin VI, but known RRL-binding partners – OPTN, TAX1BP1 and GIPC1 – failed to localize to GJ plaques. This raises the possibility that myosin VI targets to GJs by some other binding partner. Alternatively, since recent studies suggest that myosin VI harbors a second ubiquitin-binding motif, which overlaps with the RRL binding site and is disrupted by the ΔRRL mutant (He et al., 2016), it is possible that interactions with ubiquitylated substrates (including ubiquitylation sites in the Cx43 C-terminal tail) determine myosin VI localization to GJs.

Regardless of the recruitment mechanism, we observed a striking reduction in GJ plaque size when myosin VI was lost in HeLa cells stably expressing Cx43–GFP, *sv* MEFs and *sv* cardiac tissue. Furthermore, loss of myosin VI led to reduced intercellular communication. Traditionally, GJIC is assessed by scrape-loading cells with Lucifer Yellow, photobleaching calcein-loaded cells or seeding calcein-loaded cells onto a monolayer of cells (Abbaci et al., 2008). While widely used, scrape-loading requires cellular disruption and drop-in assays require normal GJ formation. The latter two techniques are single-cell assays and are laborious. Microfluidic chip strategies have been devised to obtain population-level GJIC data with minimal cellular disruption (Bathany et al., 2011), but these techniques require advanced microfluidics experience. Instead, we employed the BioPen, a highly compatible microfluidic pipette, to gently and selectively load cells with calcein AM. With this method, we measured GJIC across more than 20 cells per experiment and showed that significantly less calcein AM diffused across cell monolayers in *sv* MEFs (with equal loading confirmed by WGA staining). Given the novelty of this assay, we also conducted calcein FRAP experiments to confirm this defect.

To determine the mechanism of GJ plaque reduction with myosin VI loss, we first used a brefeldin A forward trafficking synchronization assay to show that plaque re-formation was defective in myosin VI-null MEFs. At 1 h after BFA washout, similar amounts of Cx43 had accumulated within the Golgi, but plaque growth was decreased in myosin VI-null cells. We also used the RUSH system to create a Cx43 construct that we could use to observe Cx43 forward trafficking using live-cell microscopy fully. Using the RUSH assay, we did not observe any myosin VI-dependent delays in Cx43 trafficking throughout the secretory pathway. This dispensability of myosin VI in the forward trafficking of vesicular Cx43 is consistent with reports of targeted delivery to adherens junctions by microtubules and the plus-end-tracking protein EB1 (also known as MAPRE1) (Shaw et al., 2007).

To characterize plasma membrane-localized Cx43, we used sptPALM to show that myosin VI loss did not affect Cx43 diffusional mobility in non-plaque areas. TIRF microscopy further demonstrated a selective recruitment of myosin VI to the edges of GJ plaques, and indeed, quantification of Cx43 accretion after photobleaching of plaques showed defective Cx43 plaque accretion when myosin VI was lost. These findings suggest an alternative to the prevailing endocytosis model of myosin VI function in GJ homeostasis, with myosin VI instead functioning to deliver Cx43 molecules into plaques, facilitating the established role of actin in this process (see Fig. 8).

Actin-containing microfilaments and GJs have been long observed in close proximity to each other (Larsen et al., 1979). To date, Cx43 linkages to actin include the tight junction protein ZO-1 and the actin-binding protein drebrin. Studies have shown that both dominant-negative ZO-1 and drebrin silencing by siRNA cause Cx43 loss from the intercellular junction and reduced cell communication (Butkevich et al., 2004; Toyofuku et al., 1998). Furthermore, actin polymerization inhibition by cytochalasin D has been shown to reduce dye transmission between cells and impede recovery of photobleached Cx43 GJ plaques (Martin et al., 2001; Thomas et al., 2001). Other studies have claimed that actin depolymerization also reduces plaque formation (Shaw et al., 2007).

To date, an association of hypertrophic cardiomyopathy with myosin VI loss has been described for a number of patients and two different mouse models (Avraham et al., 1995; Mohiddin et al., 2004; Williams et al., 2013). Numerous studies have investigated the expression and localization of myosin VI in skeletal muscle throughout myotube differentiation and in response to denervation, but investigations in cardiac muscle have been less robust (Karolczak et al., 2013, 2015). One study confirmed an increase in the heart-weight-to-body-weight ratio in *sv* mice, and found that the absence of myosin VI caused an increase in interstitial fibrosis that was unrelated to mouse age; however, this investigation focused on myosin VI expression in vascular endothelial cells of the heart and lungs and did not comment on myosin VI localization to the intercalated disc as previously described (Hegan et al., 2015; Karolczak et al., 2014).

In summary, this work identifies a new role for myosin VI in the construction and maintenance of GJ plaques between cells, setting the stage for further investigations into other connexin derangements. Mice lacking myosin VI had a modest reduction in the GJ intercalated disc size in their hearts, so it will be interesting to assess the maintenance of Cx43 homeostasis, including Cx43 accretion rates into GJs, in response to pathological insults such as ischemia and chronic elevations in afterload. Apart from the heart, Cx43 is also the major connexin expressed in other cell types – namely, astrocytes. The *sv* mice have been reported to display a profound reactive astrogliosis (Osterweil et al., 2005), so it will be interesting to see whether this is linked to reduced GJ function in reactive astrocytes in the brain.

## MATERIALS AND METHODS

### Reagents and antibodies

Affinity-purified rabbit antibodies against myosin VI, TAX1BP1 and optineurin were generated as previously described (Buss et al., 1998; Morriswood et al., 2007; Sahlender et al., 2005). Commercial antibodies against the following proteins were used: Cx43 [C6219; 1:200 immunofluorescence (IF), 1:2000 western blotting (WB)] and actin (A2066; 1:5000 WB) polyclonal antibodies (Sigma), Cx43 (05-763; 1:200 IF) monoclonal antibody (Millipore), GAPDH (6C5; 1:1000 WB) and clathrin (X22; 1:100 IF) monoclonal antibodies (Abcam), Dab2 (H-110; 1:100 IF) and GIPC1 (N-19; 1:100 IF) polyclonal antibodies (Santa Cruz Biotechnology) and pan-cadherin (4068; 1:1000 WB) polyclonal antibody (Cell Signaling). Phalloidin–Alexa-Fluor-647, Calcein AM, WGA–647 and Hoechst 33342 were purchased from ThermoFisher. Bafilomycin A1, brefeldin A and biotin were purchased from Sigma-Aldrich. JF_646_-HaloTag ligand was a kind gift from Joel Slaughter and Luke Lavis (Janelia Farm, HHMI, Ashburn, VA) (Grimm et al., 2015).

### Cell culture and transfections

HeLa cells were cultured in RPMI, while NRK cells (CRL-1570) and MEFs (prepared as previously described in Tumbarello et al., 2012) were cultured in Dulbecco's modified Eagle's medium (DMEM). RPE cells (CRL-2302) were cultured in DMEM:Ham's F-12 (50:50) and 30 mM sodium bicarbonate. All media were supplemented with 10% fetal bovine serum (FBS), 2 mM glutamine, 100 U/ml penicillin and 100 µg/ml streptomycin. All cell lines were periodically confirmed to be mycoplasma free by Hoechst 33342 fluorescence microscopy.

cDNA transfections were performed according to the manufacturer's instructions by using FuGENE (Promega) for HeLa cells and Lipofectamine 3000 (Invitrogen) for all other cells. For traditional calcein AM loading, cells were incubated with 0.5 μM calcein AM for 30 min before washing with complete growth medium. For brefeldin A forward trafficking assays, cells were incubated in 5 μg/ml brefeldin A for 6.5 h followed by a chase in complete medium for the indicated times. Bafilomycin A1 treatment was conducted at a concentration of 100 nM for 6.5 h. For surface biotinylation, the Pierce cell surface protein isolation kit was used according to the manufacturer's instructions. For RUSH trafficking experiments, biotin was added to the medium to a final concentration of 40 µM at the time of imaging.

For HaloTag ligand conjugation, the far-red JF_646_-HaloTag ligand (HTL) was diluted 1:200 and then 1:5 in complete medium. Cells were incubated in JF_646_-HTL-containing medium for 20 min at 37°C and washed multiple times with complete medium before imaging. Once conjugated, sample handling was identical to that for proteins tagged with fluorescent proteins as above.

Myosin VI ON-TARGET plus SMARTpool siRNA oligonucleotides (as previously described in Tumbarello et al., 2012) were purchased from Dharmacon and transfected into cells using Oligofectamine (ThermoFisher) according to the manufacturer's instructions. For efficient knockdown of myosin VI, cells were transfected on days one and three before processing on day five. Efficiency of depletion was assessed by western blotting.

### Primary culture of neonatal rat ventricular myocytes

All experiments were conducted in accordance with the United Kingdom Animals (Scientific Procedure) Act of 1986. NRVMs were established from wild-type and *Snell's waltzer* neonatal mouse pups. Surgically removed hearts were stored in ice-cold PBS and isolated ventricles were incubated in 0.5% trypsin-EDTA overnight with constant shaking. The following day, ventricles were warmed in NRVM medium (500 ml medium 199 plus 2 mM L-glutamine, 10 mM HEPES, 100 μM non-essential amino acids, 1.75 g glucose, 10 μg/ml vitamin B12 and penicillin-streptomycin) supplemented with 10% FBS and digested with type II collagenase (Sigma) (1 mg/ml in HBSS). Three collagenase fractions were added to HBSS on ice, centrifuged, resuspended and strained with a 70 μm cell strainer (Fisher). Single cells were resuspended in warm NRVM medium with 10% FBS and plated on a 10 cm dish to isolate fibroblasts. 90 min later, enriched cardiomyocytes were plated at a concentration of 10^6^ cells/well (for a 12-well dish) on fibronectin-coated coverslips (Sigma). On subsequent days, NRVMs were washed with NRVM medium supplemented with 2% FBS and imaged within 2 days.

### Plasmids

GFP–myosin-VI constructs and mutants were generated as previously described (Arden et al., 2007; Chibalina et al., 2007; Tumbarello et al., 2012). GFP–optineurin, GFP–TAX1BP1 and GFP–GIPC1 were generated as previously described (Morriswood et al., 2007; Sahlender et al., 2005). Cx43–TagRFP-t and Cx43–GFP were constructed by subcloning the respective fluorescent proteins into Cx43-mApple, a kind gift from Geoffrey Hesketh (University of Cambridge, UK). mEOS3–Cx43 was constructed by cloning Cx43 into a C-terminal mEOS3 vector, a kind gift from Prabuddha Sengupta (NICHD, NIH, Rockville, MD). HaloTag–myosin-VI was created by cloning myosin VI into a HaloTag vector C (Promega). SBP–GFP–Cx43 was created by first cloning Cx43 into a C-terminal GFP vector (Clontech) and then inserting GFP–Cx43 into a C-terminal SBP-tagged RUSH construct, a kind gift from Aubrey Weigel (NICHD, NIH, Rockville, MD). Stable cell lines were generated by amphotropic retroviral transduction of the above constructs cloned into the pLXIN retroviral vector, G418 selection and enrichment through fluorescence activated cell sorting (courtesy of the National Eye Institute Flow Cytometry Core).

### Western blotting

Cell monolayers were lysed in 2× Laemmli sample buffer and boiled for 5 min prior to SDS-PAGE using Mini-PROTEAN TGX Stain-Free precast gels (Bio-Rad). After gel activation, as per the manufacturer's instructions, proteins were transferred to a low fluorescence PVDF membrane (Bio-Rad), blocked and incubated with primary and secondary antibodies (given above). Membranes were then incubated for 5 min in the Clarity enhanced chemiluminescence western blotting substrate (Bio-Rad) and imaged on a Bio-Rad ChemiDoc. The integrated intensity of immunolabeled protein bands was calculated using Bio-Rad Image Lab software and normalized by Bio-Rad stain-free UV tryptophan labeling.

### Immunofluorescence

Cells were grown on glass coverslips (Carl Zeiss) or in Nunc Lab-Tek coverglass chambers (ThermoFisher). Cells were washed with PBS, fixed with 4% formaldehyde (Electron Microscopy Services) and permeabilized with 0.2% Triton X-100 (Sigma) in PBS. Fixed cells were blocked with 1% bovine serum albumin (BSA) before incubation with primary antibodies and detection using Alexa Fluor 488- or 568-conjugated secondary antibodies and 647-conjugated phalloidin (Invitrogen). Coverslips were mounted on slides using ProLong Anti-fade mounting medium (ThermoFisher) and Lab-Tek coverglasses were covered with PBS. Images were acquired on a Nikon Ti3 spinning disk system operated by Nikon Elements software.

Whole hearts were fixed in 4% formaldehyde and cryoprotected with 10% and 30% sucrose. Hearts were embedded in OCT medium, flash-frozen in liquid nitrogen, sectioned on a Leica CM1850 cryostat and mounted on chilled slides. Mounted heart sections were post-fixed in 4% formaldehyde, permeabilized in 0.2% Triton X-100 and blocked in 3% BSA. Blocked samples were incubated in primary antibodies at 4°C and the next day, samples were incubated in secondary antibodies and mounted on coverslips with ProLong Gold (ThermoFisher). Images were acquired on a Zeiss LSM710 confocal microscope operated by Zeiss ZEN software.

### Live-cell imaging

Cells were grown on Lab-Tek coverglass chambers, glass-bottom dishes (MatTek) or 15-mm round coverslips under normal conditions. Cells were imaged on a Nikon Ti3 spinning disk or a 3i spinning disk system. All systems were equipped with temperature regulation, while the Nikon system was also equipped with CO_2_ control. For 3i imaging, phenol red-free DMEM supplemented with 2 mM L-glutamine, 10% FBS and 20 mM HEPES was used. TIRF microscopy was conducted on a Nikon Ti3 TIRF system with an Andor EMCCD camera. For this imaging modality, #1.5 coverslips were coated with 20 μg/ml fibronectin (Millipore).

### FRAP studies

FRAP studies were conducted using a 3i spinning disk microscope and 3i SlideBook software. For GJIC studies, single cells were photobleached with 405 nm wavelength light and imaged for 10 min to measure fluorescence recovery. Calcein recovery was quantified using ImageJ. Specifically, three regions of interest (ROIs) were measured over time: the photobleached ROI, *ROI(t)*; the whole-cell ROI, *Tot(t)*; and an ROI for background fluorescence intensity, *BG(t)*. For each time point, the background intensity was removed, photobleaching was accounted for by dividing the photobleached ROI by the whole-cell ROI and each time point was normalized by the initial intensity (Eqn 1). Normalized values were plotted over time.
(1)
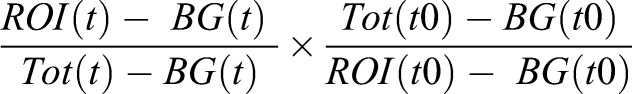
For GJ plaque FRAP experiments, a square region of a GJ plaque was photobleached with 405 nm light and plaques were imaged every 5 min for 40 min. Cx43 plaque accretion was quantified manually using ImageJ to calculate the mean pixel intensity at the growing plaque edge.

### Micropipette-assisted calcein loading

A microfluidic pipette (BioPen, Fluicell) controlled by a micromanipulation apparatus (Eppendorf) was used to selectively load a region of cells. Using an administration pressure of 200 mBar, a solution containing Alexa Fluor 647 (ThermoFisher) was first used to demarcate the fluid sphere. The micropipette was lowered until the area of dye fit inside the 60× field of view and matched that administered for other samples. The micropipette was then switched to medium containing calcein AM and WGA conjugated to Alexa Fluor 647 (ThermoFisher) and loading was imaged on a Nikon spinning disk microscope. After 10 min, the micropipette was removed and cells were incubated for 60 min in the microscope environmental chamber before a 4×4 field image was captured and stitched together using Nikon Elements Software.

### Single-particle-tracking photoactivation localization microscopy

sptPALM experiments were conducted as described previously (Manley et al., 2008). Briefly, MEFs were transfected with mEOS3–Cx43 and seeded on fibronectin-coated #1.5 coverslips for TIRF microscopy. Cells were imaged using a Nikon Ti3 TIRF system equipped with an Andor EMCCD camera. mEOS3 molecules were photoconverted using a laser intensity, pulse length and duration chosen to maintain a sparse population for localization and tracking. Measurements were taken for seven MEFs each transfected with mEOS3–Cx43. Molecules were localized using the QuickPALM ImageJ plugin and tracks were assembled using MATLAB (The Mathworks, Inc., Natick, MA). A linear fit of mean squared displacements for 250 ms trajectories with at least 15 steps was used to calculate the diffusion coefficient *D* for each condition.

### Image processing

All images were processed using the ImageJ (NIH) FIJI build and compiled in Adobe Illustrator. Live-cell images were converted into .avi movies using ImageJ and converted into .mov files using Quicktime (Apple). Custom ImageJ macro scripts were created to quantify GJ size, intensity and number using threshold-defined regions of interest (ROIs). For myosin VI GJ line profiles, 10 µm lines were drawn across all junctional structures (pointing toward the center of the cell). Channel intensity was then calculated along this line, saved and averaged with all other GJ plaques for the specified cell. For micropipette calcein-loading experiments, lines were manually drawn across the administration region and line plots were generated.

### Presentation of data and statistics

All graphs were produced using GraphPad Prism software. Bar graphs represent the mean plus the s.d. or s.e.m. as specified. Statistics were calculated using the unpaired Student's *t*-test.
